# Transversus abdominis release (TAR) procedure: a retrospective analysis of an abdominal wall reconstruction group

**DOI:** 10.1038/s41598-022-22062-x

**Published:** 2022-10-31

**Authors:** Carlos Eduardo Rey Chaves, Felipe Girón, Danny Conde, Lina Rodriguez, David Venegas, Marco Vanegas, Manuel Pardo, Ricardo E. Núñez-Rocha, Felipe Vargas, Jorge Navarro, Alberto Ricaurte

**Affiliations:** 1 School of Medicine, Pontificia Universidad Javeriana, Calle 6A #51a - 48, 111711 Bogotá D.C., Colombia; 2 Department of Surgery, Hospital Universitario Méderi, Bogotá, Colombia; 3 School of Medicine, Universidad del Rosario, Bogotá, Colombia; 4 School of Medicine, Universidad de los Andes, Bogotá, Colombia

**Keywords:** Quality of life, Risk factors

## Abstract

Complex abdominal wall defects are important conditions with high morbidity, leading to impairment of patients' physical condition and quality of life. In the last decade, the abdominal wall reconstruction paradigm has changed due to the formation of experienced and excellence groups, improving clinical outcomes after surgery. Therefore, our study shows the perspective and outcomes of an abdominal wall reconstruction group (AWRG) in Colombia, focused on the transverse abdominis release (TAR) procedure. A retrospective review of a prospectively collected database was conducted. All the patients older than 18 years old that underwent TAR procedures between January 2014–December 2020 were included. Analysis and description of postoperative outcomes (recurrence, surgical site infection (SSI), seroma, hematoma, and re-intervention) were performed. 47 patients underwent TAR procedure. 62% of patients were male. Mean age was 55 ± 13.4 years. Mean BMI was 27.8 ± 4.5 kg/m^2^. Abdominal wall defects were classified with EHS ventral Hernia classification having a W3 hernia in 72% of all defects (Mean gap size of 11.49 cm ± 4.03 cm). Mean CeDAR preoperative risk score was 20.5% ± 14.5%. Preoperative use of BOTOX Therapy (OR 1.0 *P* 0.00 95% CI 0.3–1.1) or pneumoperitoneum (OR 0.7 *P* 0.04 95% CI 0.3–0.89) are slightly associated with postoperative hematoma. In terms of hernia relapse, we have 12% of cases; all of them over a year after the surgery. TAR procedure for complex abdominal wall defects under specific clinical conditions including emergency scenarios is viable. Specialized and experienced groups show better postoperative outcomes; further studies are needed to confirm our results.

## Introduction

Abdominal wall defects are considered a complex pathology with increasing prevalence in surgical practice^1^. Patients that underwent laparotomy have a 22–55% risk of herniation^1,2^. Frequently, patients that underwent abdominal surgical procedures due to critical illness, must endure complex abdominal wall defects that directly impact their quality of life after survival^3^. Additionally, failure of hernia repair comprises between 25 and 54% of cases, resulting in an increment in costs, morbidity, and impact on quality of life^4–8^. Therefore, the need for further surgical procedures to correct abdominal wall defects, represents a challenge even for experienced surgeons, seeking a reliable, safe, and durable repair^2–4^.

Novitsky et al. 2012, presented a novel technique that modified the traditional surgical approach (Usually Rives-Stoppa procedure) for patients with important abdominal wall defects^5^. As a result, the Transversus abdominis muscle release (TAR) was proposed, showing good results in terms of recurrence, and postoperative and intraoperative complications^5^. TAR technique is based on the principal goals of an abdominal wall reconstruction: restoration of abdominal wall functionality preserving autologous tissue, and reinforcement by a durable mesh with less proportion of complications^5,6^. After the presentation of this novel technique, numerous centers started its implementation as part of their regular practice reporting similar results, such as Appleton et al. and Pujani et al.^1^, who found a low incidence of recurrence, and morbidity^1,2^.

Surgical expertise is the result of experience associated with the volume of procedures and hours of training, and the impact it may have on postoperative outcomes^9^. Relationships between surgeon volume and postoperative outcomes have been established in different studies in terms of abdominal wall hernia repair, defining low volume as < 12 hernia repairs, intermediate 12–23 procedures, high 24–35 surgeries, and very high > 36 procedures^10^. Aquina et al.^10^ found a positive association between surgeons with intermediate, high, and very high volume and a decrease in incisional hernia recurrence, postoperative complications, and hospital costs^10^. Hence, excellence centers and groups with experienced surgeons have been created through the years with the goal of achieving better results, as presented by Giron et al.^11^, this specialization may result in better outcomes^11–14^. Therefore, we present the experience of an abdominal wall reconstruction group (AWRG) outcomes with total abdominis release procedure for complex ventral hernias.

## Methods

### Study population

With the Institutional (Hospital Universitario Mayor Méderi) Review Board’s approval and following Health Insurance Portability and Accountability Act (HIPAA) guidelines, a retrospective review of a prospectively collected database was conducted. All patients over 18 years of age that underwent TAR procedures between January 2014 and December 2020 were included. Patients with no surgical description and missing data were excluded. Ethical compliance with the Helsinki Declaration, current legislation on research Res. 008430–1993 and Res. 2378–2008 (Colombia), and the International Committee of Medical Journal Editors (ICMJE) were ensured under our Ethics and Research Institutional Committee (IRB) approval.

Since 2014, in our institution, experienced surgeons (5 out of 25 staff surgeons) with an interest in open and laparoscopic procedures created a group specialized in abdominal wall pathology and reconstruction (AWRG). Also, this group usually performs 130 abdominal wall repairs per year making it a high reference and a national reference center. All the patients with abdominal wall defects (inguinal, ventral, lateral) were assessed in a multidisciplinary board including surgery, anesthesia, and nutritional support. The patient selection process was based on institutional guidelines. Measurement of hernia defect was performed based on tomography findings, incisional hernia volume, and loss of volume were calculated according to the literature^15^. The surgical risk was calculated using preoperative scores (Carolinas Equation for Determining Associated Risks (CeDAR) and NSQIP risk score of the ACS). CeDAR score is used to determine risk of wound complications and to estimate associated costs (in-hospital and follow-up charges). To estimate this, the CeDAR score uses the following variables: concurrent gastrointestinal tract entry, component separation, abdominal infection, advancement skin flap, prior hernia repair, existing stoma, BMI, uncontrolled diabetes, and tobacco use^16^.

### Preoperative botox administration

Botox applications were performed in all cases in an outpatient context and 6 weeks before the procedure. The injection was performed by interventional radiology with ultrasonographic guidance locating the external and internal oblique muscles and the transversus abdominis muscle. Injections were placed bilaterally, with 50 units for each layer (total 500 U) of type A botulinum toxin.

### Preoperative pneumoperitoneum

For these cases, a double lumen catheter was placed into the intraperitoneal space by interventional radiology with ultrasonographic guidance. The total volume was calculated according to the incisional hernia volume calculated by tomography. Pneumoperitoneum volume is 3 times the incisional hernia volume until the appearance of symptoms (abdominal pain, abdominal discomfort, or dyspnea). This was performed usually 1 week before the procedure.

The risk of wound infection was assessed based on past clinical history (malignancy, smoking, corticosteroids, and chemotherapy). Smoking habit was stopped 30 days prior to surgery for all patients. After surgical board review, the type of procedure and mesh placement was defined and explained to the patient. Patients with recurrent hernias in this study were treated extrainstitutionally and surgical data is limited. For that reason, type of mesh, mesh removal, and mesh location couldn’t be described.

### Follow-up

Preoperative data included patient demographics, comorbidities, surgical history, CeDAR score, and CT results. Intraoperative and postoperative data included surgical findings, type of mesh, type of mesh fixation, 30 days morbidity and mortality, and follow-up. In-hospital postoperative complications were assessed by the same surgeons included in the AWRG. Follow-up data at 8 days, 1, 12, 30, and 60 months was acquired, when possible, regardless of the hernia size. All patients were appointed at the same time for external evaluation. A new physical exam of all patients was performed when possible. In the follow-up meeting with the patient, a quality life survey (SF-36) was conducted, alongside a physical exam, and anamnesis to evaluate postoperative evolution, and hernia recurrence.

### Statistical analysis

Descriptive statistics were reported in terms of variable nature. Qualitative analysis was performed in terms of frequencies and percentages. In contrast, quantitative analysis was done in terms of mean and standard deviations of normally distributed data and medians and interquartile ranges (IQRs) for non-normally distributed data. Bivariate analysis was performed. Qualitative variables were analyzed using chi-square statistics (Fisher's exact test when appropriate). Quantitative variables were analyzed, based on normality, with Spearman's or Pearson's association's correlation coefficients accordingly. Bivariate analysis between qualitative and quantitative variables was performed using the Mann–Whitney test or the t-test for independent samples. For associations between categorical variables, odds ratios with 95% confidence intervals were provided. Multivariate analysis was performed, including all variables that showed association with a significant statistical value of *P* < 0.2 based on bivariate analysis, also, multivariate results were analyzed with a *P* < 0.05 cut off point.

### Surgical technique

The midline incision is performed, based on preoperative CT assessment, to avoid inadvertent enterotomy. In midline defects, dissection of the Hernial sac is performed to free it from adjacent soft tissue. Lysis of parietal adhesions is accomplished using blunt (hydrodissection) and sharp dissection. The abdominal cavity is packed with warmed compresses for visceral protection. The posterior rectus sheath is divided, on one side, lateral to the linea alba. Blunt dissection of adhesions is performed until lateral neurovascular bundles are visualized medially to the semilunar line. On the other side, the superficial pass of the hernial sac allows access to retro rectus space. Retro rectus dissection is performed as on the previous side. Falciform is dissected of línea alba and posterior rectus sheath (PRS). Medial edges of PRS are divided (extra-peritoneally) until the rostral ends at the xiphoid within the fatty triangle of Schumpelick^2^. Preperitoneal dissection is performed inferiorly to the arcuate line (Retzius space) as in classic Rives-Stoppa dissection. At the epigastrium, the muscular belly is exposed by turning the musculoaponeurotic junction of Transversus Abdominis (TA) medially. TAR begins by dividing the posterior lamina of the internal oblique, revealing TA’s fibers, which are divided using monopolar energy. The pre-transversalis plane is dissected bluntly until the psoas muscle is visualized. TA division is extended medially into the epigastrium and parallel to the musculoaponeurotic line of transition connecting to the opposite side. Reconstruction of the visceral sac is accomplished by suturing the PRS-peritoneum complex with absorbable sutures (delayed). Large polypropylene mesh (synthetic absorbable or composite mesh was used if posterior fascia was deficient) is placed extra peritoneally, anchoring one corner to the Copper's ligament. Subfascial suction drains were placed in difficult dissection cases. The anterior sheath is closed with polydioxanone 0 running suture. “U” shaped sutures were placed in places where appreciable tension was identified during closure. Redundant subcutaneous tissue and lean skin flaps were excised. The type of suture used for posterior sheath closure was Polydioxanone (PDS 1). In addition, the type of mesh used was 30 cm × 30 cm polypropylene low-density mesh that was fixed with Polypropylene (Prolene 1) cardinal knots.

### Ethical approval

All procedures performed in studies involving human participants were in accordance with the ethical standards of the institutional and/or national research committee and with the 1964 Helsinki declaration and its later amendments or comparable ethical standards.

### Informed consent

Informed consent was obtained from all individual participants included in the study, and reposes in clinical history.

## Results

### Preoperative characteristics

A total of 47 patients underwent TAR procedure for correction of complex abdominal wall defects. 63.8% of patients were male. Mean age was 55 ± 13.4 years. Mean BMI was 27.8 ± 4.5 kg/m2. History of hypertension was presented in 36.1%, T2DM in 7 patients, history of smoking in 17% of all patients and 97.8% had a surgical history with median laparotomy incision (Supplementary Table 1-See appendix).

Mean gap size of abdominal wall defects was 11.49 ± 4.03, with 74.4% of all cases classified as W3 (EHS classification) defects. 4.2% of patients had a W1 (EHS classification) defect but all of them were recurrent hernias and in two cases had concomitant lateral hernias (Supplementary Table 2-See appendix). TAR procedure was performed in an emergency context in 8% of all cases. Patients that underwent TAR procedure under emergency conditions have a 0% rate of re-interventions, and 12,5% of seroma and superficial surgical site infection. Preoperative pneumoperitoneum was indicated in 9 patients, with a mean amount of 1383 cc and a maximum of 5000 cc. Preoperative risk was analyzed by the surgical board using CeDAR score with a mean of 20.5% ± 14.5%. (Supplementary Table 2-See appendix).

### Intraoperative characteristics

Surgical mesh was used in all cases using lightweight polypropylene in 95.7% of the patients, and in 100% of the cases, we used a bare mesh. A discontinuous suture was the preferred fixation method (51%). Mean intraoperative bleeding was 101 cc ± 112.5, with mean surgical time of 225 min ± 59.9 min. Additional procedures were performed in 21.2% of cases (such as liberation of adhesions^7^ or umbilical hernia repair^3^) (Supplementary Table 3-See appendix).

### Complications and follow-up

2.1% of patients had hematoma, 4 patients presented seroma. Surgical site infection was divided into superficial (10.6%), deep (0%), and organ-related (0%). Pulmonary thromboembolism was presented in 6.3% of cases. Only 2 patients (4.2%) required surgical re-intervention: for surgical drainage of postoperative hematoma ((Supplementary Table 4-See appendix). None of our patients require mesh explant or open abdomen. In terms of hernia relapse, we have 12.7% of cases; all of them over a year after the surgery.

### Hernia recurrence

In bivariate analysis diabetes mellitus (T2DM) OR 0.75 (0.5–0.9) (*P* = 0.131), smoking habit OR 0.55 (0.3–0.7) (*P* = 0.05), and history of recurrent hernia OR 0.66 (0.2–0.8) (*P* = 0.122) were selected for multivariate analysis. Nonetheless, there was no statistically significant association found in the multivariate analysis.

### Hematoma, Seroma, Surgical site infection (SSI) and reintervention

In multivariate analysis, associated factors for hematoma in postoperative follow up were smoking OR 0.49 (0.2–0.63) (*P* = 0.02), use of preoperative Botox OR 1.0 (0.3–1.1) (*P* = 0.00), and pneumoperitoneum OR 0.7 (0.3–0.89) (*P* = 0.04). Associated factors for surgical site infection, in multivariate analysis, were diabetes mellitus OR 1.1 (0.66- 1.35) (*P* = 0.01), COPD OR 0.5 (0.25–0.65) (*P* = 0.08). In terms of surgical reintervention, in multivariate analysis, the only associated factor was chronic renal impairment OR 1.5 (0.9–1.2) (*P* = 0.001). (Supplementary Table 5-See appendix)).

## Discussion

Throughout time, complex abdominal wall defects have increased due to patients' possibility to overcome critical illness after laparotomy^17^, presenting a new surgical challenge that requires a wide armamentarium of surgical techniques and approaches, seeking to provide a proper reconstruction, assuring functionality of the abdominal wall^18^. Several advances in hernia surgery have been presented to achieve durable and safe repairs, trying to gain muscular tissue^18^. Initially, Ramirez et al.^19^ described an anterior component separation technique, nonetheless, due to the high proportion of morbidity associated with large skin flaps, other techniques were proposed. Therefore, retro muscular techniques were developed based on River-Stoppa procedure, showing a safe, and durable repair despite a high risk of neurovascular bundle damage^18,19^. Novitsky et al.^18^, to improve retro muscular repair, proposed a posterior component separation with transversus abdominis release procedure, showing positive results, with less proportion of recurrence (< 4% in 12 months), postoperative complications (hematoma (< 1%), seroma (< 3%), surgical site infection (SSI) (< 10%)) and an acceptable median length of hospitalization of 5.9 days^4,18^.

In the same line, Belyansky et al.^20^ presented a novel minimally invasive approach for Transversus Abdominis Release completely laparoscopically in 3 patients with adequate outcomes. They found that there was no need for subcutaneous drains and no perioperative complications were reported, concluding that laparoscopic abdominal wall reconstruction with transversus abdominis release could be a feasible approach that should be explored with a bigger sample size^20^. Additionally, a recent systematic review with metanalysis^21^ revealed that from 831 patients who underwent TAR, 237 underwent robotic transversus abdominis release and these patients were associated to lesser complications rate, lower risk of developing systemic complications and shorter hospital stay. But they tended to have a longer operative time compared to an open approach for TAR procedure^21^.

Regarding CeDAR, Augenstein et al.^22^ evaluated a prospective ventral hernia repair study the risks of the complications and estimated cost burden through a mathematical equation that was then converted to a novel proposed smartphone application. They found that of the 500 patients included, 17.8% developed complications and 2.2% mesh infections, with a hospital cost of 65.2 + /- 69.6 and 82.80 + /- 79.8 (in $1,000) respectively. In our study, the preoperative risk score was a mean of 20.5% ± 14.5%, a similar risk as evidenced in the literature. The previous, suggests that CeDAR score seems to be a feasible score to predict estimated cost and complications risk for clinicians to predict the risk to appropriately counsel patients^22,23^.

Several risk factors have been described for postoperative complications, and recurrence rates^24^. Factors associated with higher recurrence rates described in the literature are obesity (BMI greater than 25 kg/m2), smoking history, T2DM, corticosteroid use, and procedure performed in an emergency context^25–27^. In terms of postoperative complications such as seroma, hematoma, and SSI, a clear association has been determined with smoking history, T2DM, and COPD^28–31^. We found in our results that smoking history (OR 0.49 (0.2–0.63) (*P* = 0.02)) showed an association with the presence of hematoma. These results are in accordance with Lindstrom et al.^32^ where a clear relationship between tobacco consumption and postoperative complications was addressed. Regarding Fields et al., Hellspong et al. and Martin et al. Identified T2DM and COPD as risk factors for SSI, like the association found in our study (T2DM OR 1.1 (0.66- 1.35) (*P* = 0.01), COPD OR 0.5 (0.25–0.65) (*P* = 0.08)^30,31,33^. In terms of reintervention, Chronic renal impairment showed a positive association OR 1.5 (0.9–1.2) (*P* = 0.001), probably related to renal wound healing disturbances described in the literature^34^. Additionally in our study, the use of Botox (OR 1.0 (0.3–1.1) (*P* = 0.00)), and pneumoperitoneum (OR 0.7 (0.3–0.89) (*P* = 0.04)) have shown association with presence of hematoma. This is also related to previous literature as reported by Bueno et al.^35^ who evidenced in their study that two cases developed intraabdominal hematomas related to progressive pneumoperitoneum that could be related to the increased size and difficulty of the technique, but they did not find any complications related to preoperative administration of Botox.

Due to the complexity in the management of complex abdominal wall defects, the creation of surgical groups specialized has arisen as a feasible option seeking the achievement of better postoperative outcomes, such as morbidity, mortality, and recurrence rate reduction in these patients. Chatta et al.^36^ and Raigani et al.^37^ analyzed the impact in terms of surgical outcomes and financial burden, showing that patients who underwent treatment in high-volume specialized centers have lower rates of overall complications (9.5% vs 8%), SSI (6.2 vs 5%) and reduced in-hospital stay, with an increased financial burden (OR = 1.21)^36,37^.

Mean defect gap size was 11.40 cm ± 4.03, with a median operative time and mean intraoperative bleeding of 275 min and 101 ml, like the results described in the literature^4,18^. Postoperative complication rates may vary from 0.5 to 17%. Novitsky et al.^18^ reported 428 patients that underwent TAR procedure, 9.1% had SSI (Superficial 78%, Deep 18%), 2.8% seroma, 0.8% hematoma, and 0.8% granuloma, results like those found in our population with a slightly higher presence of seroma, but less presence of deep SSI and no cases of granuloma. (Superficial SSI 10.6%, deep SSI 0%, Hematoma 2.1%, Seroma 8.5%). Other complications were documented (Thromboembolic events 6.3% and pneumonia 2.1%), with no difference with data reported in other studies^10,18^.

The recurrence rate is considered a cornerstone in hernia surgery follow-up, leading to the invention of new methods of mesh fixation to reduce its presentation (Traditional fixation methods, self-gripping mesh, biological adhesives, fibrin glue)^38,39^; nonetheless, in our population, 95.7% of the meshes were fixated with absorbing monofilament sutures. Wheeler et al. and Mehrabi et al.^40^ described a recurrence rate, in retro muscular repair technique (River-Stoppa), from 5 to 7.3%^40,41^, Novitsky et al.^18^ reported a recurrence rate of 3.7% with TAR procedure, being these results akin to those found in our population, where recurrence rate at follow up was 12%, with a mean follow up of 35,72 months (12 months = 4%). Surgeon expertise and high/intermediate volume centers are related to better postoperative outcomes, and less financial burden in hernia surgery^9–14^.

In our study, almost 8% of the patients underwent TAR procedure in an emergency context; according to Alkhatib et al.^34^, 13,6% of patients could require re-interventions due to wound complications such as surgical site infection (13,6%), and 25,6% total reports of wound occurrences; this data is comparable with our results, in the population analyzed in our study, the re-interventions rate was 4.2% and 19.1% of wound complications (seroma and superficial surgical site infection); however, all the emergency procedures were performed by surgeons included in the abdominal wall reconstruction group.

Abdominal wall defect procedures are designed aiming to not only repair the defect but also to restore the functionality of the abdominal wall, influencing patient’s self-esteem, and emotional and mental health, being life quality an important quality indicator in hernia surgery^42–46^. In our study, life quality evaluation showed great results with 93.8 pts in change of health, 88.5 in social function, and 83.8 in role limitation (Supplementary Table 6-See appendix), measured by the -ShortForm36-^45^. Based on these results we can assert that a multidisciplinary and specialized group can offer better postoperative outcomes, reduce in-hospital costs, and have a positive impact on the life quality of our patients with complex abdominal wall defects.

Among the limitations of this study are its retrospective nature and the lack of previous studies to compare our results in terms of abdominal group versus non-specialized surgical team. Although studies have appeared in recent years regarding this topic, further prospective studies are needed to validate our results.

## Conclusion

Transverse Abdominis Release (TAR) procedure for complex abdominal wall defects under specific clinical conditions including emergency scenarios is viable. Experienced groups show better postoperative outcomes with great results in terms of life quality. According to our results, preoperative pneumoperitoneum and botox therapy prior to surgery are associated with an increased risk of hematoma, however further prospective studies with larger sample sizes are needed to confirm our results.

## Supplementary Information

Supplementary Information.

## Data Availability

The datasets generated and/or analyzed during the current study are not publicly available due to institutional data protection but are available from the corresponding author on reasonable request.
