# Supplementary material for: Transversus abdominis release (TAR) procedure: a retrospective analysis of an abdominal wall reconstruction group
Source: Sci Rep. 2022 Oct 31;12:18325. doi: 10.1038/s41598-022-22062-x (PMC9622848; doi:10.1038/s41598-022-22062-x)
Supplement: Supplementary file 1 — Supplementary Information. [file 41598_2022_22062_MOESM1_ESM.docx]

Appendix

| Supplementary table 1.  Demographics and Baseline characteristics | | | |
| --- | --- | --- | --- |
| Variable | | *n* | % |
| Gender | Male | 30 | 63.8 |
|  | Female | 17 | 36.1 |
| Past History | High Blood Pressure | 17 | 36.1 |
|  | Dibetes Mellitus    Type 2 | 7 | 14.8 |
|  | Kidney Disease | 1 | 2.1 |
|  | COPD | 3 | 6.3 |
|  | Coronary Disease | 3 | 6.3 |
|  | Thyroid    Disease | 10 | 21.2 |
|  | Smoking    history | 8 | 17 |
|  | Smoking 3 months    previously | 9 | 18 |
|  | Previous    reconstruction | 13 | 26 |
|  | Steroids    consumption | 0 | 0 |
|  | Chemotherapy in the previous 6 months. | 0 | 0 |
|  | Previous    laparotomy | 46 | 97.8 |
|  | Appendectomy | 11 | 23.4 |
|  | Nephrectomy | 2 | 4.2 |
|  | Open    cholecystectomy | 8 | 17 |
|  | Intestinal    resection. | 24 | 51 |
|  | | Mean | SD |
| Age | | 55 | 13,4 |
| BMI | | 27,8 | 4,5 |

| Supplementary table 2. Preoperative characteristics |  |  |
| --- | --- | --- |
| Variable | *n* | % |
| Botox | 2 | 4 |
| therapy |  |  |
| Previous | 9 | 18 |
| pneumoperitoneum |  |  |
| Emergency | 4 | 8 |
| surgery |  |  |
| Recurrent | 12 | 25.5 |
| hernia |  |  |
|  | Mean | SD |
| CeDAR | 20,5 | 14,5 |
| GAP (cm) | 11,49 | 4,03 |
| Volume (cc)** | 2700 | 3219 |
|  | *n* | % |
| Hernia Classification |  |  |
| M1 | 21 | 42 |
| M2 | 24 | 48 |
| M3 | 29 | 58 |
| M4 | 28 | 56 |
| M5 | 24 | 48 |
| L2 | 2 | 4.2 |
| W1 | 2 | 4.2 |
| W2 | 10 | 21.2 |
| W3 | 35 | 74.4 |

| ** Median and Intequartile range |
| --- |

| Supplementary table 3. Intraoperative Characteristics | | | |
| --- | --- | --- | --- |
| Variable | | n | % |
| Mesh | Polypropilene | 45 | 95.7 |
|  | Composed | 2 | 4.2 |
|  | Continous suture | 23 | 48.9 |
|  | Separate suture | 24 | 51 |
| Other intraoperative procedures | | 10 | 21.2 |
|  |  | Mean | SD |
| Surgery | Intraoperative Bleeding (min)** | 275 | 157 |
|  | Surgical    time | 225 | 59,9 |
|  | In-Hospital stay    (Days) | 7,4 | 6,1 |
|  | | n | % |
| Associated procedures | Liberations of adhesions | 7 | 14,8 |
|  | Umbilical hernia repair | 3 | 6,38 |
|  |  |  |  |

| Supplementary table 4. Complications and Follow-up | | | |
| --- | --- | --- | --- |
|  | Variable | n | % |
| Follow up | Follow    up (Months)* | 35,72 | 17,97 |
|  | *Relapse* | *6* | 12 |
|  | *12 Months* | *2* | 4 |
|  | *> 12 Months* | *4* | 8 |
| *Complications* | *Hematoma* | *1* | 2.1 |
|  | *Seroma* | *4* | 8.5 |
|  | *Superficial  (SSI)* | *5* | 10.6 |
|  | *Deep (SSI)* | *0* | 0 |
|  | *Organ space (SSI)* | *0* | 0 |
| *Other complications* | *Pulmonary*  *Embolism* | *3* | 6.3 |
|  | *Pneumonia* | *1* | 2.1 |
|  | *Re-intervention* | *2* | 4.2 |
| *Mean and standard deviation | | | |

| Supplementary table 5. Statistical Analysis | | | |
| --- | --- | --- | --- |
|  | Outcome | | |
|  | Relapse | | |
| Variable | OR | IC (95%) | P value |
| Diabetes Mellitus | 0.75 | 0.5-0.9 | 0.131 |
| Smoking habit | 0.55 | 0.3-0.7 | 0.05 |
| Recurrent hernia | 0.66 | 0.2-0.8 | 0.122 |
|  | Hematoma | | |
| Smoking habit | 0.49 | 0.2-0.63 | 0.02 |
| Preoperative botox | 1.0 | 0.3-1.1 | 0.00 |
| Pneumoperitoneum | 0.7 | 0.3-0.89 | 0.04 |
|  | Seroma | | |
| Emergency Context | 0.9 | 0.5-1.1 | 0.2 |
|  | SSI | | |
| Diabetes Mellitus | 1.1 | 0.66-1.35 | 0.01 |
| COPD | 0.5 | 0.25-0.65 | 0.08 |
|  | Re-intervention | | |
| Renal impairment | 1.5 | 0.9-1.2 | 0.001 |

| Supplementary table 6. Life Quality | | |
| --- | --- | --- |
| Short - Form | Mean | Standard Deviation |
| Physical function | 77.1 | 6.5 |
| Role limitation | 83.8 | 11.4 |
| Emotional limitation | 79.32 | 15.8 |
| Energy limitation | 81 | 14.8 |
| Social function | 88.5 | 12.34 |
| Pain | 92.3 | 7.19 |
| Over    all health | 89.32 | 12.6 |
| Change in health | 93.8 | 7.7 |
